# Visualizing Metal Nanoparticle Electrochemical Dissolution Atom by Atom

**DOI:** 10.1002/smll.73677

**Published:** 2026-05-20

**Authors:** Pei Zhao, Daniel Houghton, Richard Beanland, Julie V. Macpherson

**Affiliations:** ^1^ Department of Chemistry University of Warwick Coventry UK; ^2^ Department of Physics University of Warwick Coventry UK

**Keywords:** atoms, boron doped diamond, dissolution, electrochemistry, gold nanoparticles, transmission electron microscopy

## Abstract

Gaining insight into the early stages of the electrochemical dissolution of metal nanoparticles (NPs) provides crucial insights into mechanisms that control this important process. Being able to do this under conditions where atom loss from individual NPs can be quantified is especially challenging. Here, we use identical‐location, annular dark field, scanning transmission electron microscopy (IL‐ADF‐STEM) to provide “time‐stamped” snapshots of the dissolution of gold NPs on electron‐transparent carbon electrodes. Dissolution is carried out in aqueous chloride (mM) solutions, at anodic potentials, over millisecond timescales. IL‐ADF‐STEM analysis of the integrated image intensities is employed to estimate the number of atoms within each NP, allowing atom loss (and gain) to be tracked for the same NP, over time, on a particle‐by‐particle basis. 3D reconstruction of NPs enables changes in 3D morphology to be visualized. Hemispherical‐shaped gold NPs ≤4 nm in diameter are interrogated, with the smallest showing the largest atom loss. NPs are revealed to flatten during dissolution, as opposed to a gradual reduction in diameter, and the number of isolated gold atoms on the surface increases. Considerable interactions between NPs also occur, including the formation of single atom bridges and coalescence events. The vertical growth of NPs is also observed.

## Graphical abstract

The early stages of the electrochemical dissolution of gold nanoparticles (NPs) on electron beam transparent carbon‐based supports are investigated using identical location scanning transmission electron microscopy at atom resolution. Numbers of atoms lost (and gained), along with morphology changes are tracked for the same NP within an ensemble, over time, on a NP‐by‐NP basis.

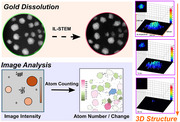

## 1 Introduction

Electrodeposition is widely used for the production of metal nanoparticles (NPs) [1, 2, 3, 4] on low energy surfaces, e.g., carbon, where particles are favoured over thin film formation. Metal NP electrodes have found extensive use as electrocatalysts for a range of applications, e.g., oxygen evolution [5], hydrogen oxidation [6], carbon dioxide reduction [7], *etc*. Given such interest, much work has been devoted to understanding the growth mechanism of NPs formed via electrodeposition on these surfaces. Early studies focused on extracting parameters such as NP size, density, growth rate and mechanism indirectly from current‐time (*i‐t*) transients averaged over the whole electrode surface [8, 9]. With the advent of high resolution microscopy techniques that can monitor NP size, density and growth rates, these models were revealed to be incomplete, and often inaccurate, descriptors of the early stages of electro‐nucleation and growth [10, 11].

Interestingly, there has been far less work which has considered the anodic dissolution mechanism, especially using methods with resolution sufficient to resolve atom‐scale processes. For the commonly used electrocatalytic metals, platinum [12] and gold [13, 14], the anodic dissolution process is intrinsically linked with the formation of a metal oxide layer [15]. On‐line inductively coupled plasma mass spectrometry (ICP‐MS) investigations in acidic media using gold electrodes, led to the hypothesis that at potentials greater than the onset potential for oxide formation, place‐exchange between O/OH and gold takes place resulting in anodic dissolution [15, 16]. Gold dissolution can be further enhanced by moving into the potential region for water oxidation/oxygen evolution [17]. Such understanding is of importance, especially when the system is operated under potential conditions sufficient to initiate dissolution of the metal [18, 19, 20]. Accessing high oxidation potentials during voltammetric cycling is also commonly used for electrochemical cleaning, or activation, of metal NPs [21, 22].

There has been relatively little work carried out on the dissolution of gold in NP form and much of the literature has focused on voltammetric cycling (anodically and cathodically) using carbon supports [15, 21, 23, 24]. Under these conditions, dissolved gold has also been detected cathodically, due to reduction of the gold oxide formed anodically [15]. Monitoring of gold dissolution has been carried out using a variety of methods including ICP‐MS [15, 24], electron microscopy (EM) [21] and electrochemical assessment of the change in the charge associated with gold oxide reduction [21]. Using identical location (IL)‐transmission (TEM) and IL‐scanning (SEM) it has also been possible to map the same NPs before and after cycling [23]. These studies and others [21] have revealed the importance of size, with gold NPs < 5 nm shown to dissolve preferentially, compared to the larger NPs. However, as resolution has been limited in the microscopy studies to date [21, 23], it has not been possible to discern the atom‐scale mechanisms by which the gold NPs dissolve.

Anodic dissolution can be enhanced for gold via the addition of chloride or bromide ions, with higher dissolution rates typically found as halide ion concentrations increase. Direct anodic dissolution of gold is possible due to the formation of soluble gold‐halide complexes [25]. Most studies have been carried out using gold electrodes at halide concentrations > mM, where gold loss is monitored using techniques such as ICP‐MS [25], electrochemical quartz crystal microbalance [26, 27], and the rotating ring disc electrode [28]. Ultra‐high resolution studies have been carried out using electrochemical (EC) scanning tunnelling microscopy on gold single crystal electrodes where at high enough potentials dissolution from both terrace and step sites is evident [29].

Voltammetric studies on size‐specific chemically synthesized gold NPs tethered to electrode surfaces have shown that for NPs < 4 nm in diameter, in bromide media, the electrochemical dissolution potential of gold shifts to more negative values, and is well separated from that due to gold oxide formation [30], as NP size decreases. This is attributed to an increasing surface energy [31]. Work on larger gold NPs using in‐situ EC‐TEM and scanning electrochemical cell microscopy have also highlighted the dynamic nature of the oxide passivation/dissolution process [32]. In situ EC‐TEM provided direct evidence for pitting of the passivation layer, exposing the underlying metallic gold core to halide ion assisted dissolution [32].

In this work, we visualize the electrochemical dissolution of hemispherical‐shaped gold NPs of diameters ≤4 nm, at the single atom level with millisecond temporal resolution on a conducting boron‐doped diamond (BDD) support [10, 33], using high‐resolution ex situ IL‐aberration‐corrected scanning TEM (STEM). This is combined with image analysis (atom counting and 3D image rendering) to estimate the change in the morphology and the number of atoms lost (or gained) per NP, on a particle‐by‐particle basis as a function of time. Previous IL‐EC‐TEM studies have provided valuable insights into electrochemically induced changes in Pt alloy NPs [34, 35], but quantitative analysis was restricted to the facets of an individual NP that happened to be perfectly aligned to the electron beam, producing a lattice image where atom columns could be resolved. Here, using estimated atom counts based on integrated intensity and particle footprint in ADF‐STEM images, we are able to analyse the full population of NPs in the image window, independent of alignment with the electron beam. In this way we elucidate the mechanisms associated with the early stages (0 – 15 ms) of electrochemically induced gold NP dissolution in the presence of mM chloride, at a level commensurate with atom level descriptions [34].

## 2 Results and Discussion

### 2.1 Characterization of Gold NPs on the BDD TEM Substrate

The BDD TEM electrode, for use in ex situ IL‐TEM studies is shown in Supporting Information 1, SI 1, Figure S1. It is produced [33] as described in SI 1, and contains a small hole in the centre of the electrode, about 50–100 µm in diameter, around which the BDD is electron beam transparent. At the edge, the BDD thickness is typically less than 50 nm, measured using electron energy loss spectroscopy [33]. The “coastline” of the hole edge has recognizable features, which act as useful location guides to aid IL imaging. The BDD has superior mechanical and electrochemical properties for IL‐TEM imaging compared to conventional carbon film TEM grids [36].

**FIGURE 1:**
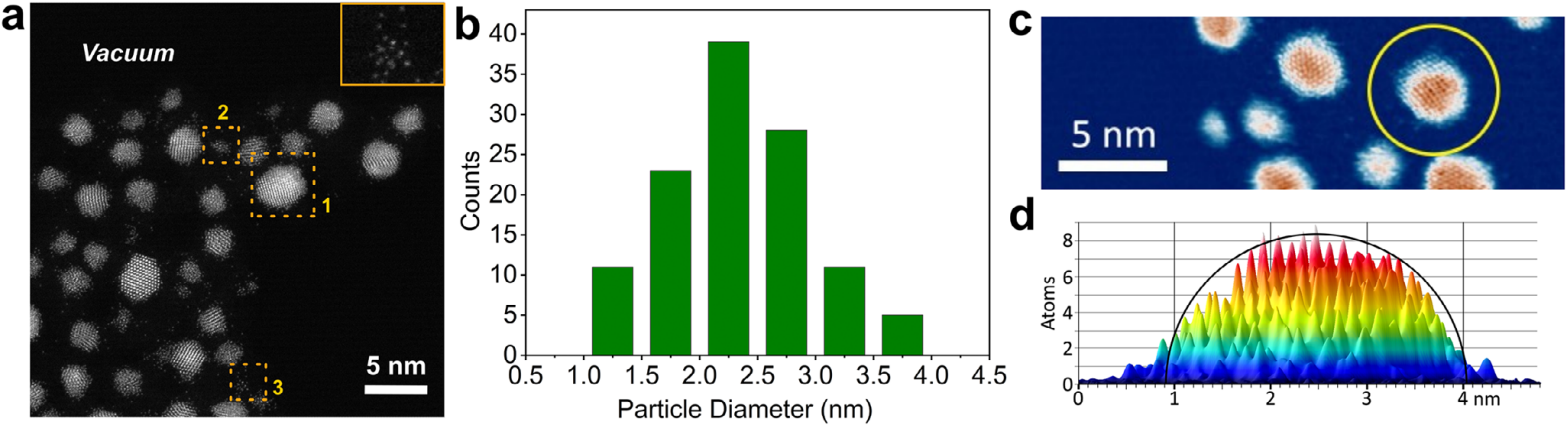
(a) Typical ADF‐STEM image of gold NPs (including atom clusters) and single atoms on the BDD TEM electrode in the area of i in Figure S1. (b) Size distribution of gold NPs from a and Figure S2a,b. (c) A region of the background‐subtracted image showing the gold NPs. (d) 3D rendering of the gold NP in the yellow circle in (c) with y axis displaying number of atoms per atom column; the NP is shown to be hemispherical.

Gold NPs on the BDD‐TEM surface were deposited by sputtering (SI 2). Figure 1a shows a high resolution annular dark field (ADF)‐scanning TEM (STEM) image of the gold NPs on the BDD TEM substrate taken in the electron transparent region, marked (i) in Figure S1 with a dashed green square. The hole region of the BDD appears black. Figures S2a,b show two other regions, further demonstrating that in all areas of the BDD‐TEM electrode the gold takes two forms: (1) NPs, e.g. as shown by the dotted orange box, labelled 1, in Figure 1a. We also include atom clusters under the term NP (*vide infra*), these show no defined arrangement and are typically smaller in size, e.g. dotted orange box labelled 2, Figure 1a.  (2) Isolated single gold atoms (e.g., dotted orange box, labelled as 3, Figure 1a, and also shown in the inset at higher resolution). Single gold atoms on the (110) surface of BDD (appropriate crystal orientation for the BDD used herein [33]) have been theoretically predicted to be stabilized by surface defects and/or boron [37].

**FIGURE 2:**
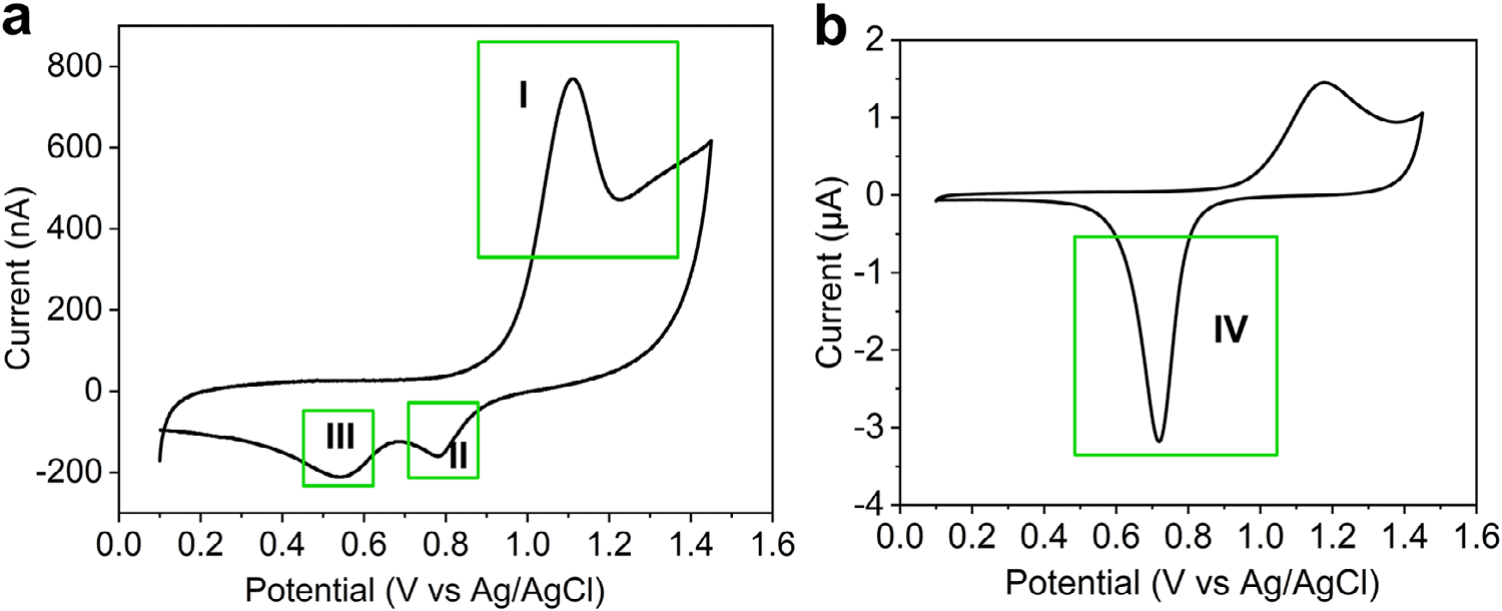
(a,b) CV of gold NPs on a BDD disk electrode (1 mm diameter) at a scan rate of 100 mV s^−1^. The electrolyte is (a) 4 mM KCl and 0.1 M HClO_4_ (b) 0.1 M HClO_4_ only.

A histogram showing the distribution of gold NP diameters, for NPs ≥ 1.0 nm, is given in Figure 1b, for the NPs in Figure 1a, Figure S2a,b (*n* = 117). The gold NPs have sizes in the range 1.0 – 3.9 nm, with an average diameter of 2.4 ± 0.6 nm. The images shown are representative of gold NP size distributions across the BDD‐TEM electrode. Figure 1c shows a background‐subtracted (*vide‐infra*) image of gold NPs. The NP in the yellow circle shows a clear lattice pattern. 3D rendering of this NP is shown in Figure 1d, with the vertical axis given in number of atoms. The process of converting ADF‐STEM intensity to atoms is described in SI 3. The gold NP with the intensity profile shown in Figure 1d, can be best described as a hemisphere and has a maximum height of seven atoms and contains ∼630 atoms. Further experimental confirmation of NP geometry is provided by Figure S7, SI 4, which shows a cross‐sectional TEM image of several Au NPs, taken from a lamellae cut from the sample using a focused ion beam lift‐out procedure. Hemispheres have also been found to be the predominant geometry for gold NPs formed by thermal evaporation on a carbon support [38].

### 2.2 Electrochemical Measurements

Experimental details on solutions used, the experimental set‐up and electrochemistry performed are provided in SI 5a. Figure 2a shows a typical first scan cyclic voltammogram (CV) for a gold NP BDD disk macroelectrode (1 mm diameter) recorded in 0.1 M HClO_4_ and 4 mM KCl at a scan rate of 100 mV s^−1^ over the potential range 0.10–1.45 V vs. Ag/AgCl. In the positive‐going potential sweep, the anodic current begins to rise at 0.90 V vs. Ag/AgCl resulting in a broad peak (I) which falls rapidly before starting to rise again slowly. This peak likely represents dissolution of gold in the presence of Cl^−^ (Equations 1 and 2) [26, 39], with the slowly rising current at more positive potentials indicative of passivation of the surface due to the formation of a gold oxide layer (Equation 3) [26, 29].

(1) $$Au+4Cl^{-}\to AuCl_{4}^{-}+3e^{-}\;$$

(2) $$Au+2Cl^{-}\to AuCl_{2}^{-}+e^{-}\;$$

(3) $$Au+H_{2}O\;\to AuO+2H^{+}+2e^{-}\;$$

Figure 2b, shows the CV response for oxidation of the gold NPs in a halide free acid solution (0.1 M HClO_4_). Oxidation of the gold NPs, Equation (3), occurs at more positive potentials compared with gold dissolution in the presence of Cl^−^, Equations ((1), (2)), confirming the interpretation of the CV in Figure 2a.

During the negative‐going potential sweep in Figure 2a, two cathodic peaks are observed. The peak (II) at +0.78 V vs. Ag/AgCl is attributed to the reduction of gold oxide which formed during the anodic sweep. The peak (III) at +0.55 V vs. Ag/AgCl is associated with the re‐deposition of dissolved gold species, which can form during either anodic dissolution or reduction of gold oxide [26, 27]. Such features have been observed previously during gold electrochemical dissolution in acid solution containing chloride ions [25, 26]. When chloride ions are removed from solution only one peak is observed, Figure 2b, indicating the reduction of gold oxide, peak (IV).

To investigate the very early stages of gold NP electrochemical dissolution, accelerated by chloride ions, a constant potential was applied to the gold NP‐BDD TEM electrode over a milliseconds timeframe. From the CV measurement in Figure 2a, the potential was stepped from 0.8 V vs. Ag/AgCl, where no faradaic processes occur, to a high anodic potential of 1.4 V vs. Ag/AgCl to ensure dissolution. The experiment was first carried out for 5 ms, and then repeated for a further 10 ms; this gives a total time spent at 1.4 V vs. Ag/AgCl of 15 ms. Prior to dissolution and after each potential pulse, IL‐ADF‐STEM was carried out to observe changes to the gold NPs on the BDD‐TEM substrate and quantify them in terms of atom number, size and shape, on a particle‐by‐particle basis. Experimental details for IL‐ADF‐STEM are described in SI 5b. *i‐t* transients are depicted in Figure S9a, b, SI 6. The currents observed over 0−5 ms and 5−15 ms decay exponentially with time and contain contributions from gold dissolution and oxide formation. Non‐faradaic decay of the current due to electrical double layer charging is expected at timescales < ms, for a BDD TEM electrode of the size employed herein [33].

### 2.3 Image Analysis and Estimates of Atom Count

Given metal NPs can be studied at the atomic scale by electron microscopy [40], the number of constituent atoms in a NP is an obvious metric of interest. Various approaches for atom counting have been suggested [41, 42, 43, 44, 45], although they almost all rely on images of NPs almost perfectly aligned to the electron beam, in which atom columns are well‐resolved. Tomographic methods have been attempted [46, 47, 48], but this greatly increases electron dose and computational requirements, often meaning that only a single NP is analysed [47].

In our data, many of the NPs do not show a clear lattice pattern, due to not being aligned to the electron beam, and the smaller gold clusters have no regular structure. Integrated ADF‐STEM intensity (i.e., the background‐subtracted electron intensity summed over an entire NP) is thus used here as a metric to measure the number of atoms present per NP. This approach sacrifices some experimental precision in comparison with counting atoms in perfectly aligned NPs, but brings the advantage of the ability to analyse large numbers of NPs with arbitrary orientations, using only single images. For this approach to be viable three conditions must be met: the NP integrated intensity must be proportional to atom count; the constant of proportionality must be determined and; any background intensity must be removed. Below, we briefly outline the main steps, with more detailed information given in SI 3.

To establish the relationship between atom count and intensity, multi‐slice simulations of gold crystals in several different orientations were performed (with imaging conditions corresponding to our experiment), Figure S3a. The different crystal orientations do have differences in intensity but they are relatively small, giving a typical error of ca. 5%. Figure S3b shows that the integrated intensity is well approximated by a linear relationship, up to fifteen atoms in an atom column. Taking the volume of a gold atom to be that of the Wigner‐Seitz cell of bulk fcc gold, a hemispherical NP of height 15 atoms has a diameter of ca. 9 nm. This is considerably larger than any of the NPs in Figure 1, or observed during any stage of the dissolution process. Thus, a linear relationship between the integrated intensity of a NP, and the number of atoms it contains, is a valid approximation for our data.

Quantification of experimental ADF‐STEM intensities can be performed by careful calibration of the annular scintillator detector relative to the incident beam intensity [49]. Here, we find that a useful calibration can be obtained using only the experimental images of NPs with intensity profiles consistent with a hemispherical shape. For these NPs we can obtain a second estimate of atom count, independent of their integrated intensity, simply from their volume. We thus obtain a constant of proportionality between intensity and atom count by equating these two estimates, averaged over many measurements, as shown in Figure S4, SI 3. To reflect the degree of uncertainty in our measurements we give atom counts, determined by hemispherical volume and integrated intensity, as estimated values rather than precise numbers. As will be apparent from the results presented below these uncertainties do not prevent the observation of many interesting phenomena in gold NP dissolution.

The BDD TEM support, although electron transparent, still contributes a small amount of background intensity that varies across the image and prevents the use of a simple threshold to isolate the gold NPs. To account for this, the background intensity profile was estimated by excluding the gold NPs and inpainting with edge intensities propagated and smoothed over the missing part of the image, (SI 3, Figure S5b). Justification for this approach is given in SI 3, Figure S6. The generated background was subtracted from the original image, leaving an image with intensities due only to the gold atoms and NPs, Figure S5c.

### 2.4 Identical Location Measurements of Atom Loss/Gain Per NP

**FIGURE 3:**
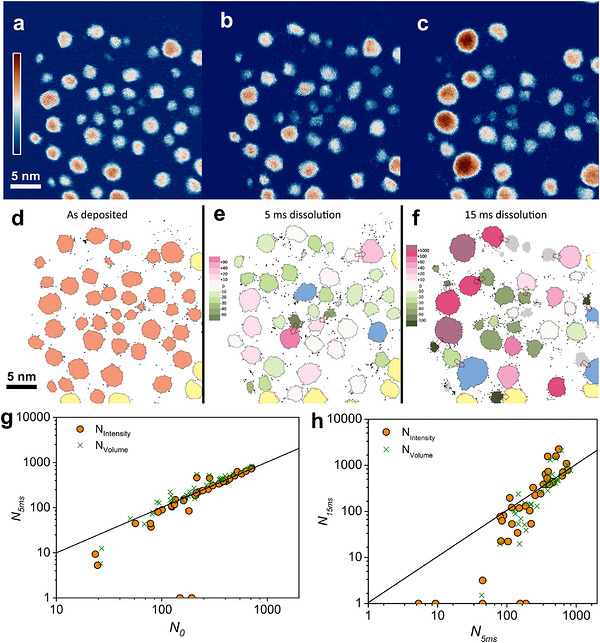
(a‐c) The background subtracted IL‐ADF‐STEM images of gold NPs at 0, 5 and 15 ms dissolution times. (d‐f) The segmented gold NP analysis of IL‐STEM images. Colour coding, as‐deposited: orange = counted, yellow = excluded (edge); other images blue = merged, green = shrinking, red = expanding, with contrast proportional to the change in atom count. NPs that disappear completely are shown in grey. (g, h) Scatter plot of atom number changes in each NP over the dissolution series, shown on a logarithmic scale. The black line indicates no change. (g) From as‐deposited to 5 ms dissolution and (h) from 5 ms to 15 ms dissolution. Circles = atom count estimated from integrated intensity (method 3) and crosses = estimated from (assumed hemispherical) volume (method 2). NPs that disappear or merge with others are shown at the horizontal axis.

Three IL‐ADF‐STEM background subtracted images for anodic dissolution times of 0, 5 ms and 15 ms, are shown in Figure 3a,b and c respectively. No carbon contamination was observed when imaging. The raw IL‐ADF‐STEM images are shown in Figure S10a–c, SI 7. Outlines depicting the boundaries of these structures, across the three images, are shown in Figure S10d–f, SI 7. Two distinct types of gold structures were tracked using identical location: NPs (where we also include atom clusters in this category) and isolated single atoms. For each NP, the total number of atoms, *N_x_*, per dissolution time frame (where *x* is the time, i.e., *N_0ms_*, *N_5ms_* and *N_15ms_*) was estimated and changes in response to dissolution time quantified. The total number of single atoms and NPs were also counted for each image (see Table S1, SI 7).

To help visualize changes in atom number for each individual NP as a function of time, the IL‐ADF‐STEM images in Figure 3a–c, are colour coded, as shown in Figure 3d–f. NPs that have lost atoms are coloured **green** and those that have gained atoms are coloured **red**; with increasing contrast indicating a greater change in atom number (compared to the preceding image). Atom counting was undertaken using Method 3 in SI 3. **Blue** NPs are formed due to merging. **White** NPs indicate a size change ≤5% of the preceding image atom count, and NPs that disappear completely are shown in **grey**. The image taken at 0 ms, Figure 3d, shows the original state of the NPs prior to dissolution, those that are orange are counted, whilst those that are yellow are excluded as they intersect an edge of the image.

As Figure 3e shows, after 5 ms dissolution, many of the NPs display a low green contrast indicating only a small loss in the number of atoms. In contrast, after 15 ms dissolution, Figure 3f, some NPs have dissolved completely (compared to the 5 ms image and marked in grey) and several NPs have merged to form larger ones. Qualitative numerical visualization of atom loss/gain is shown in the plot in Figure 3g,h on a logarithmic scale, which estimates the number of atoms in each NP from as‐deposited to 5 ms dissolution (Figure 3g) and 5 ms to 15 ms dissolution (Figure 3h). The black line indicates no change; points below this line indicate NPs that have lost atoms between measurements, while points above show NPs that gain atoms (including NPs that merge, in which case the change is assigned to the larger NP before merging). The relative lack of change for many NPs is clear in Figure 3g. However, this is not true of the smaller NPs, where larger changes are observed, with the two smallest NPs (*N*_0_ < 30 atoms) losing a significant percentage of their constituent atoms (i.e., well below the black line). Two mid‐sized NPs are lost by merging, and they appear on the horizontal axis. Also plotted, as crosses in Figure 3g, are the number of atoms estimated assuming hemispherical NP shapes (method 2 in SI 3).

An interesting observation, is the growth of a small number of NPs during anodic dissolution in Figure 3e. After a further 10 ms of anodic dissolution (total time = 15 ms), significant changes in NP size are visible, with large NPs growing and smaller ones shrinking (data points above and below the line respectively in Figure 3h). The data in Figure 3h also shows that it is predominantly the large NPs (after 5 ms dissolution), containing more than ∼300 atoms, which grow. In a small number of cases some NPs which lost atoms after 5 ms, start growing after 15 ms and *vice versa*. Data from another area of the BDD electrode shown in SI 8, Figure S11, shows similar trends.

### 2.5 Higher Resolution (Atom Level) Insights Into Early‐Stage Gold NP Dissolution:

#### 2.5.1 Single Atoms

Table S1 in SI 7 shows that during anodic dissolution the number of isolated single gold atoms almost doubles after the first 5 ms of dissolution (∼228 to ∼432), decreasing to ∼352 after 15 ms. Gold adatom mobility on the surface of gold in the presence of chloride is thought to be accelerated [50, 51]. However, DFT simulations show that surface defects and surface/sub‐surface boron atoms on the (110) surface of BDD [33, 52] stabilize any gold atoms that are formed [10, 37].

**FIGURE 4:**
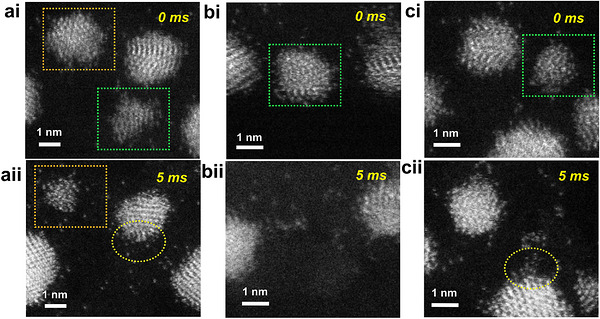
IL‐ADF‐STEM “time‐stamped” images of gold NP dynamics during electrochemical dissolution over 0 to 5 ms from three different locations (a, b and c).

The higher magnification IL‐ADF‐STEM images in Figure 4a–c highlight the possible mechanisms by which these single atoms arise over the time period 0 (i, top row) to 5 ms (ii, bottom row) of anodic dissolution. In Figure 4a–c the NPs labelled with the green dotted box have dissolved, leaving behind isolated atoms. In Figure 4cii, the remaining atoms appear to be preferentially funnelled toward the NP at the bottom of the image (indicated by the yellow dotted oval). The NP in Figure 4aii, which sat the closest to the dissolved NP, also has a higher number of single atoms associated with the lower half (yellow dotted oval). The NP labelled with the orange dotted box in Figure 4ai has decreased in size during 5 ms of anodic dissolution. The resulting structure at 5 ms, is more diffuse, especially at the edges [53] where single atoms again appear to be evident.

#### 2.5.2 Gold NP “Time‐Stamped” Dissolution Behaviour

**FIGURE 5:**
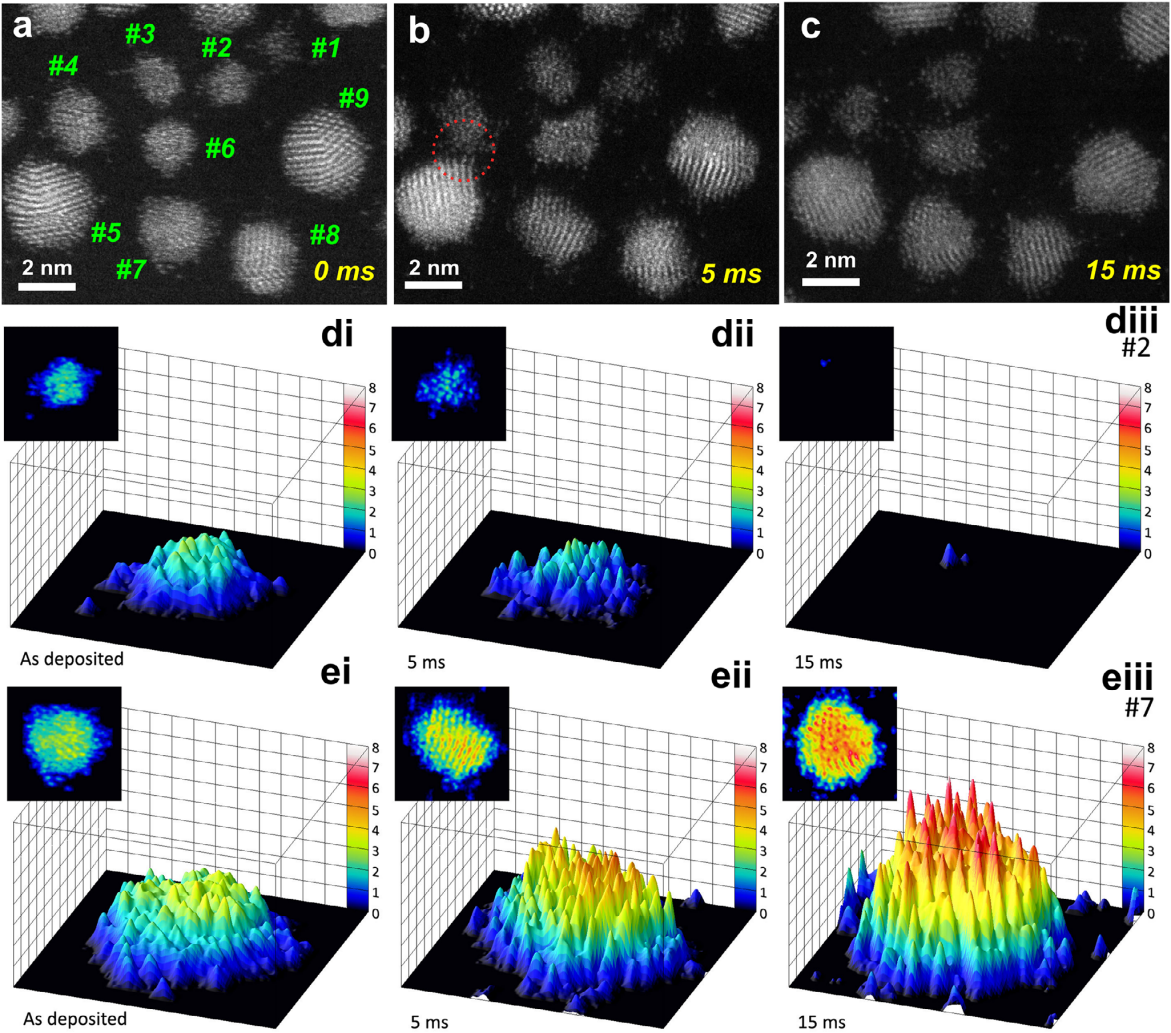
IL‐ADF‐STEM “time‐stamped” images of gold NP dynamics during electrochemical dissolution over 0 to 15 ms. Electrochemical dissolution was carried out by applying + 1.4 V vs. Ag/AgCl in chloride‐containing solution for (a) 0 ms, (b) 5 ms, and (c) 15 ms. 3D representation of the morphology changes of NP #2 (di‐iii) and NP #7 (ei‐iii) in terms of atom loss (and gain). 3D plot image width is 4.8 nm; grids on back and side wall have spacings of 0.64 nm.

Figure 5 shows the “time‐stamped” dynamics of nine different sized NPs during electrochemical dissolution over a 15 ms time period with IL images recorded at (a) 0 ms; (b) 5 ms and (c) 15 ms. The number of atoms for each NP in Figure 5a is provided in Table S2, SI 9. The two smallest NPs in terms of atom numbers, NPs #1 and 2, show the largest % change in atom loss with time. Both, at time = 15 ms, have disappeared. After 5 ms, NP #1 contains only *N_5ms_* ∼ 9 atoms, too small to even form a well ordered structure (smallest gold ordered structure contains 13 atoms [54, 55]) and the atoms instead cluster together.

Using the same procedure as employed in Figure 1d and described in SI 3, we show a 3D representation of NPs #2 and #7 as a function of time, Figure 5d,e respectively, with the *y* axis representing NP height in numbers of atoms. NP #2 is initially comprised of *N_0ms_* ∼80 atoms and has diameter 1.7 nm (Figure 5di). After 5 ms it has lost ∼40 atoms with no significant change in its footprint on the BDD surface (Figure 5dii). This highlights the preferred dissolution of lower co‐ordination gold atoms at the apex and edges of the NP, associated with higher activity. Preferential dissolution from the apex and edges has also been observed for larger palladium and platinum nanocubes subject to electron beam induced dissolution via radiolysis‐induced oxidising agents in liquid cell TEM [53, 56]. However, whilst in the case of the cubes they evolve into spheres as the dissolution process proceeds, here the NP flattens with time, and the contact area remains largely unchanged. This emphasizes the role the gold‐BDD surface interaction must be playing. After 15 ms, only one gold atom remains in the place where NP 2# resided (Figure 5diii), most likely marking a defect site in the surface [37].

In contrast when comparing the numbers of atoms before and after anodic dissolution (see Table S2, SI 9) there does not appear to be any significant change (within 5%) for NPs #7, #8 and #9 over 0–5 ms. These NPs (with the exception of NP #5) contain the largest number of atoms and appear the most stable. This is not surprising since larger size NPs, with a smaller surface area to volume ratio, should have lower associated surface energy, which makes them more stable during the initial stages of dissolution [57, 58]. After a further 10 ms NP #8 has fewer atoms compared to the 5 ms data, NP #9 has remained stable, whilst NP #7 has grown. The latter is also true of NP #5 which after 15 ms showed an increased number of atoms compared to the starting structure.

A 3D representation of the changes to NP #7 from 0 to 15 ms, is shown in Figure 5ei–iii. Interestingly, whilst the estimated number of atoms does not change significantly from 0 to 5 ms, the shape of the NP alters, becoming more hemispherical. This could be caused by rearrangement of its structure in response to the high applied anodic potential and presence of the chloride ions. From 5 to 15 ms, the NP grows in size. Figure 5eiii indicates that the NP is growing predominantly via vertical addition of atoms promoting upward growth.

Particle growth during electrochemical dissolution is perhaps unexpected, given the electrode is biased at a potential to favour dissolution (and oxide formation). There are two factors which could be responsible for this process. First, electrochemical Ostwald ripening, either via solution or mediated by the surface [57, 59]. Theoretical and experimental studies have shown that the redox potential associated with dissolution of metal nanoparticles decreases with decreasing size [31, 59, 60]. Thus, smaller metal NPs are more easily oxidised (dissolved) than larger NPs (as also demonstrated experimentally in Figure 5). The dissolution of the smaller gold NPs causes the concentration of gold‐halide complexes to locally increase around neighbouring NPs, which could drive redeposition on the more thermodynamically stable (larger) particles. Second, the impact of the open circuit potential (OCP) should be considered. After each pulse of anodic dissolution the potential of the electrode is returned to OCP (measured at 0.43 V vs. Ag/AgCl) for times less than ∼ 200 s, prior to removal of the electrode from solution. Based on the CV in Figure 2a this value is negative enough to drive redeposition. Possible OCP induced changes are discussed further in SI 10, where IL‐ADF‐STEM experiments are carried out in the presence of 10 nM AuCl_4_^−^ in 4 mM KCl and 0.1 M HClO_4_ solution. The AuCl_4_^−^ concentration was based on the value determined for dissolved gold in solution using ICP–MS, after anodic dissolution was complete. Under these conditions, IL‐ADF‐STEM experiments showed only limited evidence of Au NP growth, (SI 10), although we note this bulk concentration may not reflect the initial local concentration when the electrode first returns to OCP.

For NPs aligned with the electron beam, crystallographic changes can also be identified. For example, NPs # 5, #8 and #9 in Figure 5a all start with a twinned (decahedral) structure at 0 ms but transform to a single crystal [61, 62] after 15 ms. The structural changes associated with NP #5 are also worthy of further attention. Particle #5 grows by ∼124 atoms from 0 ms to 5 ms, seemingly by direct interaction with NP #4, which loses ∼96 atoms. Small lines of atoms emerge from the top of NP #5, and orientate toward NP #4, outlined by the red circle in Figure 5b. Atom bridges have been observed previously between gold NPs during electrodeposition and were thought to aid the movement of gold atoms between neighbouring sites [10]. Gold atom movement across bridges will be further accelerated in the presence of chloride given there is the possibility for Au‐Cl bond formation [51]. Atom bridges have also been seen using heating induced gold NP coalescence in TEM [63]. Another example of atom bridge formation is shown in SI 9, Figure S12c.

**FIGURE 6:**
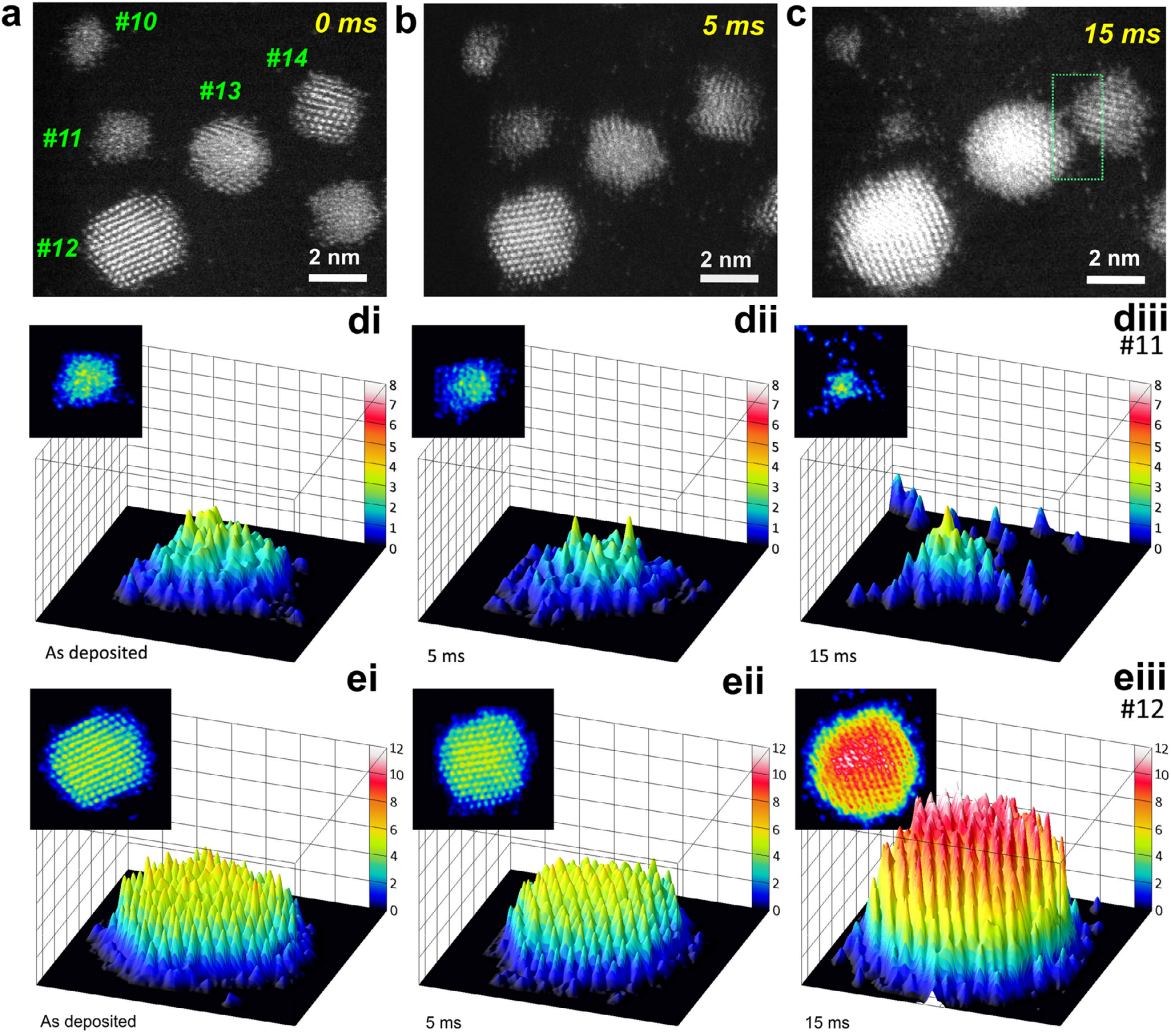
IL‐ADF‐STEM “time‐stamped” images of gold NP dissolution dynamics during electrochemical dissolution over 0 to 15 ms. Electrochemical dissolution was carried out by applying +1.4 V vs. Ag/AgCl in 4 mM chloride‐containing solution for (a) 0 ms, (b) 5 ms, and (c) 15 ms. 3D morphology changes of NP #11 (di‐iii) and NP #12 (ei‐iii). 3D plot image width is 4.8 nm; grids on back and side wall have spacings of 0.64 nm.

Further insights into the gold NP dissolution process can be obtained by consideration of the IL‐ADF‐STEM images recorded in another area of the BDD‐TEM electrode. Five gold NPs, #10‐14, at (a) 0 ms, (b) 5 ms and (c) 15 ms are shown in Figure 6; estimated numbers of atoms for each NP are given in Table S3, SI 9. The smallest NPs #10 (*N_0ms_* ∼ 92) and #11 (*N_0ms_* ∼ 125) lose ∼69 and ∼103 atoms respectively over 15 ms. As can be seen in Figure 6di–iii, NP #11 shows similar behaviour to NP #2 in Figure 5 and flattens with time. An increased number of single atoms is seen in the gap between the two NPs, especially after 15 ms. In contrast, the largest NP, #12, grows by ∼385 atoms from 0–15 ms and undergoes a similar transformation to that of NP #7 in Figure 5ei–iii, where atoms are added vertically with only a small change in base diameter (∼3.5 to 4 nm), Figure 6ei–iii. Gold atom bridge formation is again evident, here between NPs #13 and #14 at *t* = 15 ms, where NP #13 grows (adding ∼314 atoms) becoming much closer in distance to NP #14.

**FIGURE 7:**
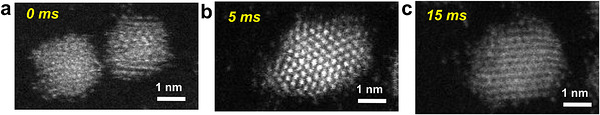
IL‐ADF‐STEM “time‐stamped” images of gold NP dynamics during electrochemical dissolution over 0 to 15 ms (a) 0 ms, (b) 5 ms and (c) 15 ms. Electrochemical dissolution was carried out by applying + 1.4 V vs. Ag/AgCl in chlorine‐containing solution.

In some instances, for very closely spaced NPs, coalescence is observed. For example, Figure 7a, shows two NPs with *N_0ms_* ∼243 (left) and ∼215 (right) respectively and a distance between them only about a single atom in size. At this distance, electrical double layers will overlap, and electron tunnelling and potential‐induced atom migration becomes possible. After 5 ms of anodic dissolution, the two NPs have become one crystalline NP, with *N_5ms_* equal to the sum of the individual NPs within estimate (*N_5ms_* ∼461 atoms, vs. *N_0ms_* ∼243+215 ∼ 458), Figure 7b. The NP exhibits an fcc structure. No dissolution has taken place, instead the gold atoms in the NP have undergone coalescence in order to minimize surface energy. Whilst an atom bridge(s) is not observed, this is a likely consequence of capturing dynamic events at discrete time intervals only. After 15 ms, Figure 7c, the NP still retains its crystalline structure but contains slightly less atoms, *N_15ms_* ∼424 atoms, indicating a small amount of dissolution. Further examples of NP coalescence are given in SI 11, Figure S15.

## 3 Conclusion

The use of IL‐ADF‐STEM imaging on conducting carbon substrates, enabled atom level information to be revealed on electrochemical, early stage dissolution mechanisms of metal NPs. Whilst STEM image analysis models were developed for gold NPs of diameter ≤4 nm, they are also applicable to larger NPs, up to 9 nm diameter, and different metals. The models, which used the integrated image intensities, facilitated estimation of the number of atoms per NP, on a NP‐by‐NP basis, for ten's of NPs in an ensemble, without the need to have the NPs aligned to the electron beam. The number of atoms lost (or gained) per NP, as a function of time, were assessed and rendered images produced to visualize 3D changes in NP morphology. Over the investigated timescale whilst many of the ∼ hemispherical shaped Au NPs showed small changes in atom number, it was the smallest NPs which displayed the largest loss, attributed to having the highest surface energies. STEM image analysis showed that dissolution proceeded preferentially at the apex of the NP, resulting in a gradual flattening, as opposed to the NP gradually reducing in diameter and height. Isolated single gold atoms remained pinned to the surface, which resulted in the numbers of single atoms increasing during dissolution. Such behavior highlighted the importance of the Au atom‐electrode interaction. Under anodic dissolution conditions, gold NP particle growth was also observed. This was attributed to either electrochemical driven Ostwald ripening or OCP driven growth. At the dissolution potentials employed whilst chloride was used to accelerate gold dissolution, we cannot rule out gold oxide formation also playing a role. Future IL‐ADF‐STEM studies will explore the impact of substrate defect (boron) density and surface chemistry on dissolution, alongside dissolution under conditions where only halide‐driven or oxide‐driven dissolution needs be considered. It will also be of interest to explore the role of NP geometry vs. substrate interaction, moving from hemispheres to spheres and cubes. Atomic level “time‐stamped” dynamic information was also captured showcasing the importance of single atom alignment, atom bridge formation between very closely spaced NPs, coalescence and atom addition. Such information will aid understanding and help further reveal the role of substrate, NP size/morphology and NP‐NP spacing in atom scale dissolution processes.

## Conflicts of Interest

The authors declare no conflicts of interest.

## Supporting information




**Supporting File**: smll73677‐sup‐0001‐SuppMat.pdf.

## Data Availability

The data that support the findings of this study are openly available in Warwick Research Archive Portal at http://www.wrap.warwick.ac.uk/200730.
