# Supplementary material for: Visualizing Metal Nanoparticle Electrochemical Dissolution Atom by Atom
Source: Small. 2026 May 20;22(34):e73677. doi: 10.1002/smll.73677 (PMC13275038; doi:10.1002/smll.73677)
Supplement: Supplementary file 1 — Supporting File: smll73677‐sup‐0001‐SuppMat.pdf. [file SMLL-22-e73677-s001.pdf]

Visualizing Metal Nanoparticle Electrochemical Dissolution Atom by Atom

Pei Zhao, Daniel Houghton, Richard Beanland*, Julie V. Macpherson*

Department of Chemistry, University of Warwick, Coventry, CV4 7AL, UK

Department of Physics, University of Warwick, Coventry, CV4 7AL, UK

*j.macpherson@warwick.ac.uk; r.beanland@warwick.ac.uk

Supporting Information

SI 1. Fabrication procedure for the BDD-TEM electrode

SI 2. Sputtered gold NPs on the BDD-TEM electrode

SI 3. ADF-STEM image analysis to enable atom counting within gold NPs

SI 4. FIB Cut Cross Sectional TEM of Au NPs on BDD

SI 5. Experimental details-electrochemistry and TEM

SI 6. Current-time transients of gold NPs electrochemical dissolution

SI 7. Raw IL-ADF-STEM images of gold NPs and their outlines

SI 8. Another set of data showing segmented IL-ADF-STEM images of gold NPs and atom size changes

SI 9. Estimated numbers of atoms for gold NPs

SI 10. Possible impacts of OCP on gold NP growth

SI 11. Further examples of gold NP coalescence

SI 1. Fabrication procedure for the BDD-TEM electrode

BDD-TEM Electrode Fabrication: All BDD used for the electrochemical studies was doped with boron (>10^{20} B atoms cm^{-3}) so that the material was above the metallic threshold,[1] and was provided by Element Six Ltd., Oxford, UK. It was grown using microwave chemical vapor deposition (CVD) such that it was thick enough (> 400 μm) to be removed from the non-diamond substrate and then both sides mechanically polished on a resin bonded wheel to produce free-standing BDD ~50 μm thickness, with a surface roughness of ~ nm. The BDD was grown under conditions to result in minimal sp^2 carbon (material of a quality equivalent to electrode E in reference 1). The BDD was cut into disks of diameter 3 mm in order to fit into the TEM holder, using laser micromachining. Electron beam transparency was achieved by Ar^+ ion milling (GATAN PIPS II). This process has been described in full in reference 2 and results in BDD disks which are concave in shape, reducing in thickness from the edge to center, where a hole has formed, as shown in Figure **S1**. The area around the hole, typically a distance of ~ 3 μm from the hole edge, is of sufficient thickness to be electron transparent.[2]

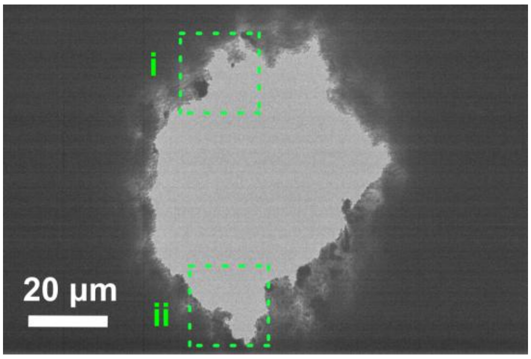

Figure S1. STEM image of the electron transparent area around the hole in the BDD-TEM substrate.

The BDD displays a dominant (110) crystal orientation.[2] The BDD TEM electrodes were acid cleaned after ion milling to remove any debris or graphitic material. An electrical contact was produced by laser roughening a small segment near and up to the edge, which also results in non-diamond carbon formation.[3] Carbon ink (MG Chemicals, 838AR) was painted onto the lasered area to help make the electrical contact between the BDD TEM electrode and fine metal tweezers.[2] The glass-sealed BDD macroelectrode (1 mm in diameter)[4] was fabricated from the same BDD material as used for the TEM substrates.

SI 2. Sputtered gold NPs on the BDD-TEM electrode

Gold NPs were sputter coated onto the BDD TEM substrate using a NanoPVD sputter system (Moorfield Nanotechnology, UK) with a 15% direct current power supply unit (DC PSU) and pressure 8 × 10^{-3} mbar, for 1 s. A gold target (99.99% purity, Moorfield) was employed. The electrical contact region of the BDD substrate was masked using a glass slide to avoid gold coating during sputtering. Figure S2a and S2b display typical ADF-STEM images of gold nanoparticles (NPs) on the BDD-TEM surface.

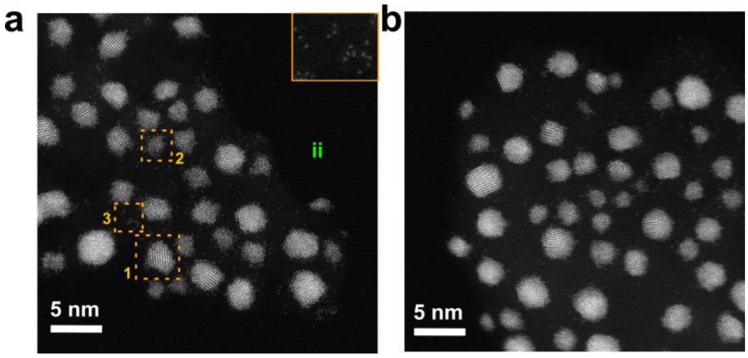

Figure S2. (a, b) ADF-STEM images of different regions of the BDD-TEM electrode showing the presence of gold NPs, box 1, including atom clusters, box 2 and single atoms, box 3 and inset. The region shown in (a) is located in area ii in Figure S1.

SI 3. ADF-STEM image analysis to enable atom counting within gold nanostructures

3.1 Three image analysis methods

Image analysis was conducted using home-written scripts and ImageJ software.

(a) Method 1

Our aim in analyzing the ADF-STEM images is to obtain an estimate of the number of atoms present in every nanoparticle (NP) in the image. We considered three possible ways of making this estimation. First, the number of distinct atom columns in a NP can be counted and in combination with making an assumption on NP shape it is possible to estimate how many atoms are in each column. However, this method only works for NPs perfectly aligned to the electron beam showing a clear lattice pattern. Hence this method could not be used on all NPs in the TEM image and was therefore discounted.

(b) Method 2

Given that most NPs have an intensity profile consistent with a hemispherical shape, a second approach is to measure the observed NP area ($A = \pi r^2$), calculate a hemispherical volume ($V = \frac{2}{3}\pi r^3 = \frac{2}{3}\frac{A^{3/2}}{\pi^{1/2}}$), and divide this result by the volume of a single atom in crystalline gold ($0.01695\ nm^3$).[5] This method only gives a good estimate N_{volume} of the number of atoms in hemispherical NPs, but the difference between this method and that of method 3 when applied to all NPs also gives useful information about NP shape.

(c) Method 3

This involves measuring the total intensity of a NP in the ADF-STEM image. This method requires: (1) a good measurement of the total NP intensity; and (2) a model of electron scattering from gold NPs that links intensity to the number of atoms present. For the latter, we performed multi-slice simulations of gold crystals in various orientations to find the relationship between total ADF-STEM intensity and the number of atoms in a column. In ADF-STEM the intensity of a column of atoms increases monotonically with an increasing number of atoms. In general, the increase is not linear and depends on electron beam energy, the material, the microscope and its optical system. To evaluate the uncertainties introduced by these aspects we performed multi-slice simulations of gold crystals in different orientations for the microscope and imaging conditions used, as shown in Figure S3a. While there are differences in intensity for the different orientations, they can be regarded as small giving an

error of, at most, one atom for small thicknesses. Our simulations show that for columns with less than 15 atoms, there is a linear relationship with intensity (R^2= 0.997), Figure S3b, and since all of the observed NPs have diameters less than 30 atoms wide, this method can be applied to all NPs irrespective of size or shape. This means that the number of atoms that comprise a gold NP can be estimated simply by measuring its integrated intensity in the ADF-STEM image.

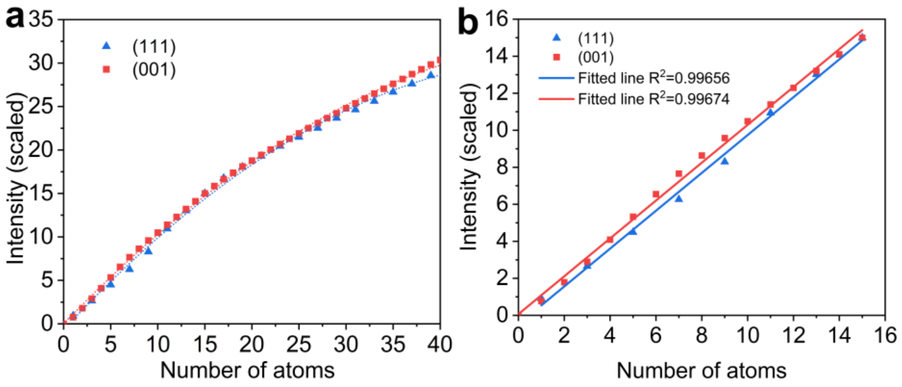

Figure S3. (a) ADF-STEM intensities of atom columns in gold crystals with orientations of [001] and [111] parallel to the electron beam calculated using a multi-slice simulation for 200 keV electrons (convergence angle 20 mrad; annular detector from 45 to 180 mrad; Cs = 1 μm). The thermal displacement parameter was chosen to be that of bulk Au, Biso = 0.5 Å^2.[6] Intensities are scaled so that the (001) simulation has an intensity of 15 for a column of 15 atoms. (b) The linear relationship between scaled intensities and columns with less than 15 atoms.

While this establishes a linear relationship between intensity and number of atoms $N = kI$, the constant of proportionality must be determined. Thus the final step in the analysis requires the measured intensity of NPs in the ADF STEM images to be scaled to match the linear relationship in Figure S3b. Specifically, a NP of integrated intensity I contains a number of atoms $N_{intensity} = I/k$, where k is a constant of proportionality. We perform this scaling by comparing integrated intensities of NPs with the number of atoms N_{volume} obtained by assuming a hemispherical shape (method 2). We then choose a value of k such that a plot of N_{volume} vs $N_{intensity}$ has a gradient of unity, as shown in Figure S4a for the 44 NPs in the image of Figure S5. As can be seen in Figure S4b there is an excellent correspondence in the number of atoms estimated by both methods. This agrees with the observation that the majority of NPs have an intensity profile consistent with a hemispherical shape. The difference between N_{volume} and $N_{intensity}$ was ± 10% (one standard deviation).

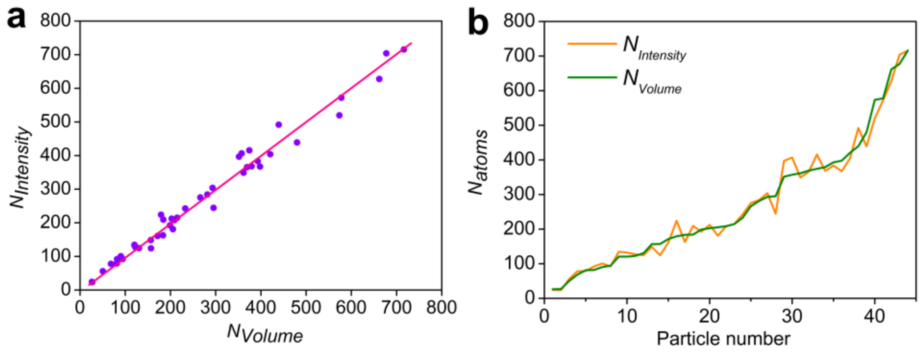

Figure S4. (a) A plot of N_{volume} vs $N_{intensity}$ with intensity scaling to give a gradient of unity. (b) Plot of the number of atoms in the 44 as-deposited gold NPs in Figure S5 estimated by both the volume and intensity methods, using the scaling shown in (b).

While scaling against the whole ensemble is appropriate for images of the hemispherically-shaped sputtered NPs, it is clearly less so for those NPs that grew in size after application of a dissolution potential for 5 ms and 15 ms. Here, only a selection of the larger NPs appeared to have hemispherical shapes and therefore scaling of $N_{intensity}$ to match N_{volume} was applied only for these NPs. This allowed the atom numbers for all particles to be estimated using $N_{intensity}$. As a caveat, due to the many unquantifiable uncertainties, in all the above methods, our calculation of the number of atoms in each NP should be regarded as an estimate rather than a quantitative measure. However, these estimates are sufficient to provide useful and interesting insights into the dissolution process.

All images were processed prior to particle analysis, as illustrated using the ADF-STEM image in Figure S5a. First, a threshold was manually selected, which formed a binary mask corresponding to the whole image excluding the centre of all large gold NPs. This mask was then eroded to exclude the periphery of the NPs and applied to a copy of the image with a gaussian blur of radius 50 pixels. This gives a smooth background of the vacuum and the BDD support in regions without NPs. The gaps in this image were then in-painted using a script that propagated the intensity at their edges to the centre (averaging from three neighbouring edge pixels until the hole is infilled), giving an estimate of background intensity between and underneath the NPs as shown in Figure S5b. This background was then subtracted from the original image, smoothing was applied using a gaussian blur of 1 pixel and the mean intensity in the vacuum region was set to zero. The result is an image with a uniform background of value close to zero that can be used for conventional particle analysis using threshold segmentation (Figure S5c).

For the images presented in the main text, the threshold was set such that NPs and single atoms

could be selected in the processed image whilst excluding background noise. Manual segmentation was used for a small number of NPs that were almost touching. Variations in intensity are illustrated in Figure S5d and e, which show a crystalline gold NP and a single gold atom close to the edge of the BDD support. The corresponding intensity profiles correspond to the raw and processed images, highlighting successful removal of the background. The variations in intensity correspond to the varying number of atoms within the NP as a function of position.

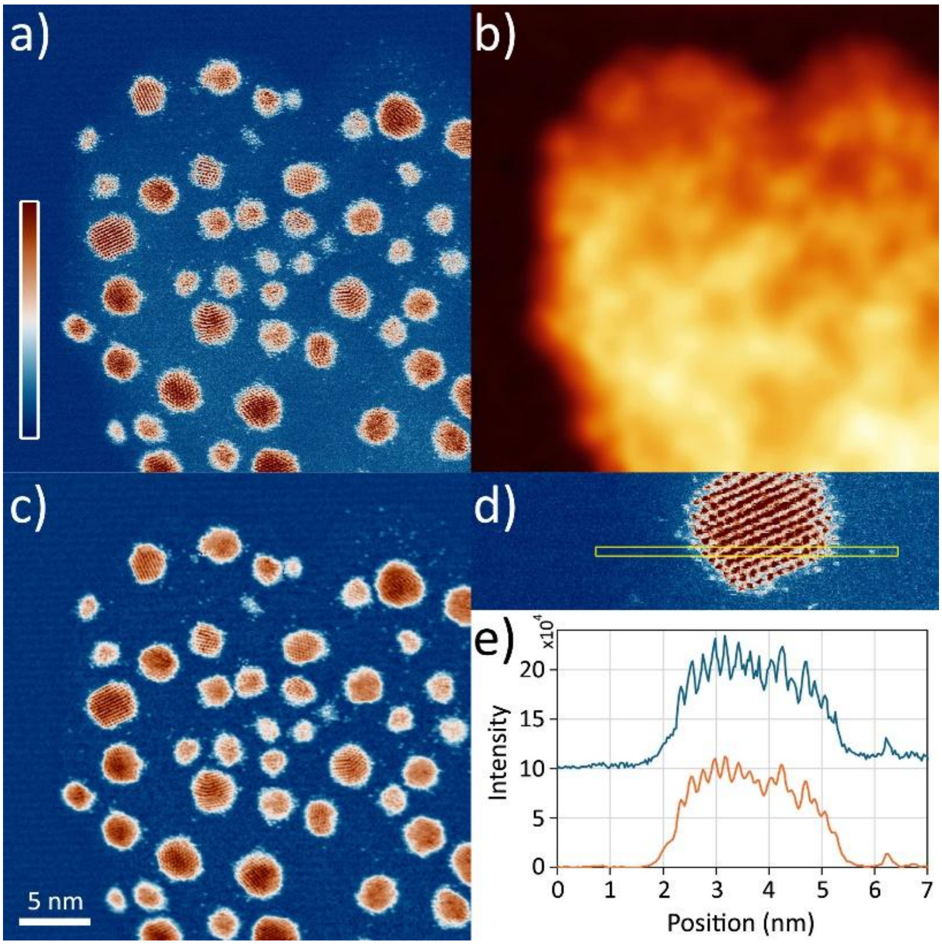

Figure S5. (a) Raw ADF-STEM image of sputtered gold NPs on a BDD support. A false colour look-up table is used to emphasise low intensities. (b) Intensity profile of the BDD support, obtained by excluding the gold NPs and inpainting. (c) The processed image after subtracting the BDD intensity, smoothing with a Gaussian blur of radius 1 pixel, and setting the vacuum level to zero. (d) A region of the image showing a gold NP and single gold atoms on the BDD support. (e) Intensity profiles along the yellow box in (d) for the raw image (blue line) and processed image (orange line).

Figure S6 shows a region from the centre of Figure 3 (main text) in which a NP dissolves completely between (a) 5 ms and (b) 15 ms. (c) shows the corresponding intensity plot from the yellow rectangle in (b). In the region of the NP which has now dissolved, the intensity

relating to the BDD background is shown to the same as that away from the NP. This shows that the thickness of the BDD support is unchanged in the position formerly occupied by the NP. This is not surprising given we have shown previously via electron energy loss spectroscopy that the BDD thickness does not change even under higher potential for long periods in high concentration acid.[7] This confirms that a simple inpainting of intensity from the BDD between NPs, interpolating between intensities on opposite edges, should provide a robust measurement of the background intensity under the NP. Since the noise in the background BDD intensity is well below that of a single Au atom (Figure S5e) we also do not expect background subtraction to have any measurable effect on the integrated intensity of Au NPs.

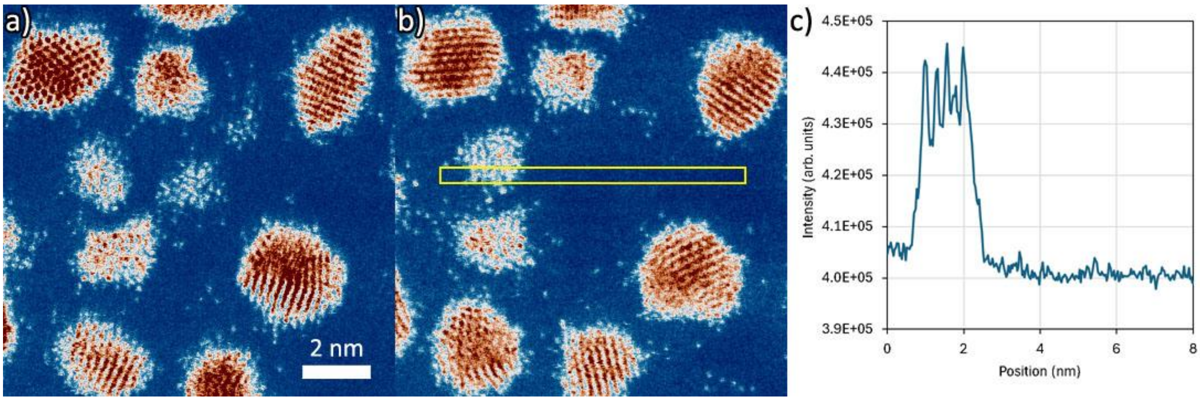

Figure S6. A region from the centre of Figure 3 in which a NP dissolves completely between (a) 5 ms and (b) 15 ms. (c) an intensity plot from the yellow rectangle in (b) showing the thickness of the BDD support is unchanged in the position formerly occupied by the NP.

3.2. 3D renderings of NPs (Figs. 1d, 5 and 6)

The 3D renderings of NPs are simply three-dimensional plots of the image intensity, made using the ImageJ 'Interactive 3D surface plot' command. They are not, and cannot be, a true atomic-scale 3D model of a NP, except in cases where they are clusters of widely spaced individual atoms. However, they provide a qualitative view of the NP and give an understanding of atom loss or addition through the time series that is very useful. Since we have a calibrated ADF-STEM intensity that gives a good match for the estimates $N_{intensity}$ and N_{volume} for hemispherical NPs, we may use the calculated height of a hemispherical NP to convert image intensity into 'atom height' which we use as the vertical scale in Figures 1d, 5 and 6 (main text). The resulting conversion factor can be applied to the whole image, allowing height scales to be applied to NPs that are not hemispherical.

SI 4. FIB Cut Cross Sectional TEM of Au NPs on BDD

A freshly sputtered gold NP-BDD TEM electrode sample was coated with ~200 nm of carbon using an Emitech K950X evaporator, in order to prove protection to the gold/BDD interface. A TEM lamella was then produced in the Tescan Amber FIB-SEM, using the standard FIB lift-out technique[8] perpendicular to the central hole in the electrode and 10 µm from the hole edge. Milling was performed at 30 keV, with subsequent polishing stages at 5 keV and 2 keV. Figure S7 shows an ADF-STEM cross-sectional image (top) of the lamella. The gold NPs on the BDD surface are clearly visible. To aid visualization, the perimeter of the gold NPs have been highlighted using different colours (bottom).

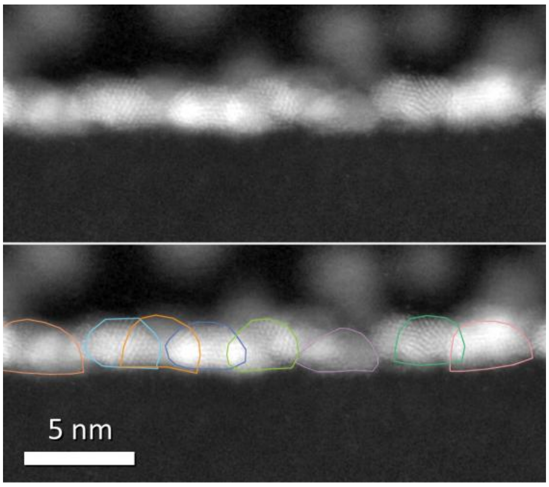

Figure S7. TEM image of the Au NP-BDD cross section (FIB cut lamellae)

SI 5. Experimental details-electrochemistry and TEM

(a) Solutions and Electrochemical Measurements: All solutions were made using 18.2 MΩ cm ultra-pure deionised water (Milli-Q, Millipore). Gold dissolution solutions contained 4 mM potassium chloride (KCl, 99.99%, Sigma-Aldrich) and 0.1 M perchloric acid ($HClO_4$, 99.99%, Sigma-Aldrich). Acid cleaning solutions for the BDD contained concentrated sulfuric acid (H_2SO_4 95−97%, Scientific and Chemical Supplies Ltd.) and potassium nitrate (KNO_3 99.0%, Scientific and Chemical Supplies Ltd.) All chemicals were used as received without further purification.

All electrochemical experiments were carried out using a three electrode set-up controlled by a potentiostat (Ivium CompactStat, Holland). Either a BDD TEM electrode or a 1 mm diameter BDD disk electrode served as the working electrode, a Ag/AgCl non-leak, ~3.5 M KCl (WPI) was used as the reference electrode and a platinum coil served as the counter electrode. A schematic of the three-electrode set-up is shown in Figure S8. Before all experiments the solution was deaerated for 30 min with nitrogen. CV of the sputtered gold NPs on the BDD 1 mm diameter disk electrode was recorded in 0.1 M $HClO_4$ with 4 mM KCl at a scan rate of 100 mV s^{-1} from 0.10 to 1.45 V vs Ag/AgCl.

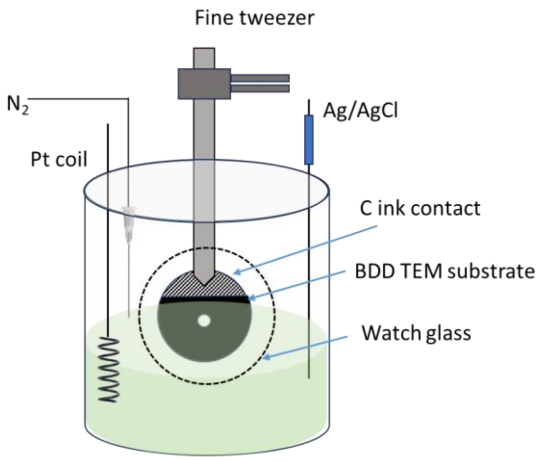

Figure S8. Schematic of the three electrode set-up

Electrochemical oxidation/dissolution was conducted in the same solution using the BDD-TEM electrode at a potential of 1.40 V vs Ag/AgCl (3.5 M). For all electrochemical measurements the BDD TEM electrode was dipped into solution, such that just over half the electrode was immersed in solution and the central hole was fully immersed (Figure S8). The potential was stepped from 0.80 V, where no faradaic processes occur to 1.40 V vs Ag/AgCl (3.5 M) for a period of 5 ms. After 5 ms the potential was returned to OCP and the BDD-TEM

electrode removed from solution, rinsed very gently with ultra-pure water and left to dry in a desiccator prior to IL-ADF-STEM imaging. The BDD-TEM electrode was then returned to the solution and the potential stepped from +0.80 V to +1.40 V vs Ag/AgCl (3.5 M) for a further 10 ms. IL-ADF-STEM images were recorded in the exact same position as for those recorded for the first 5 ms potential pulse. This resulted in three sets of IL-ADF-STEM images, 0 ms (pre-pulse), 0 to 5 ms and 5 to 15 ms (15 ms of anodic potential in total).

(b) IL-ADF-STEM: STEM imaging was undertaken in defined regions using a double-corrected JEOL JEM-ARM 200F TEM, operated at 200 kV. Typically, at least three areas were recorded to ascertain patterns of behavior (for identical location). All IL imaging was carried out in ADF-STEM mode with a fine-imaging probe at a current of ~ 23 pA (convergence semi-angle of ~ 25 mrad and an ADF detector inner angle of 50 mrad). Prior to electrochemical analysis the surface of the gold NP coated BDD was imaged using IL-ADF-STEM. It was previously shown that under similar current imaging conditions, for isolated single gold atoms on a BDD-TEM support, three consecutive IL-ADF-STEM images resulted in no negligible change in gold atom position.[9] Thus for these experiments we expect electron beam induced isolated atom movement to be minimal.

SI 6. Current-time transients of gold NP electrochemical dissolution

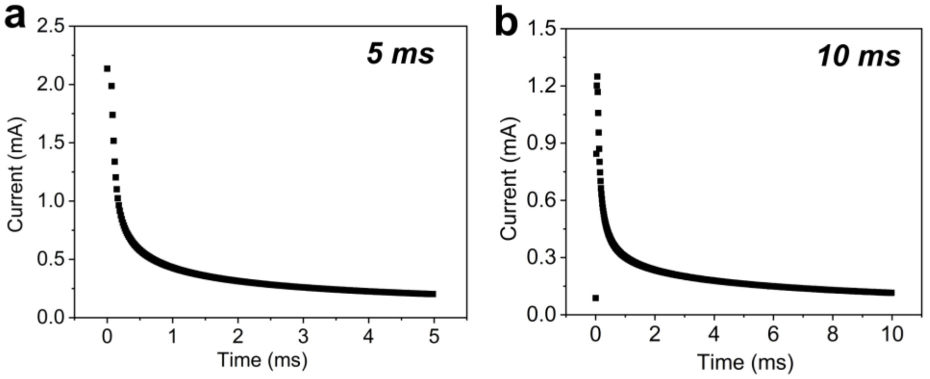

Figure S9. Current-time transients obtained for electrochemical dissolution of gold NPs during an anodic pulse applied to the BDD TEM substrate of 1.40 V vs Ag/AgCl (starting from 0.80 V vs Ag/AgCl) for (a) 5 ms and then (b) a further 10 ms (total anodic dissolution time = 15 ms).

SI 7. Raw IL-ADF-STEM images of gold NPs and their outlines

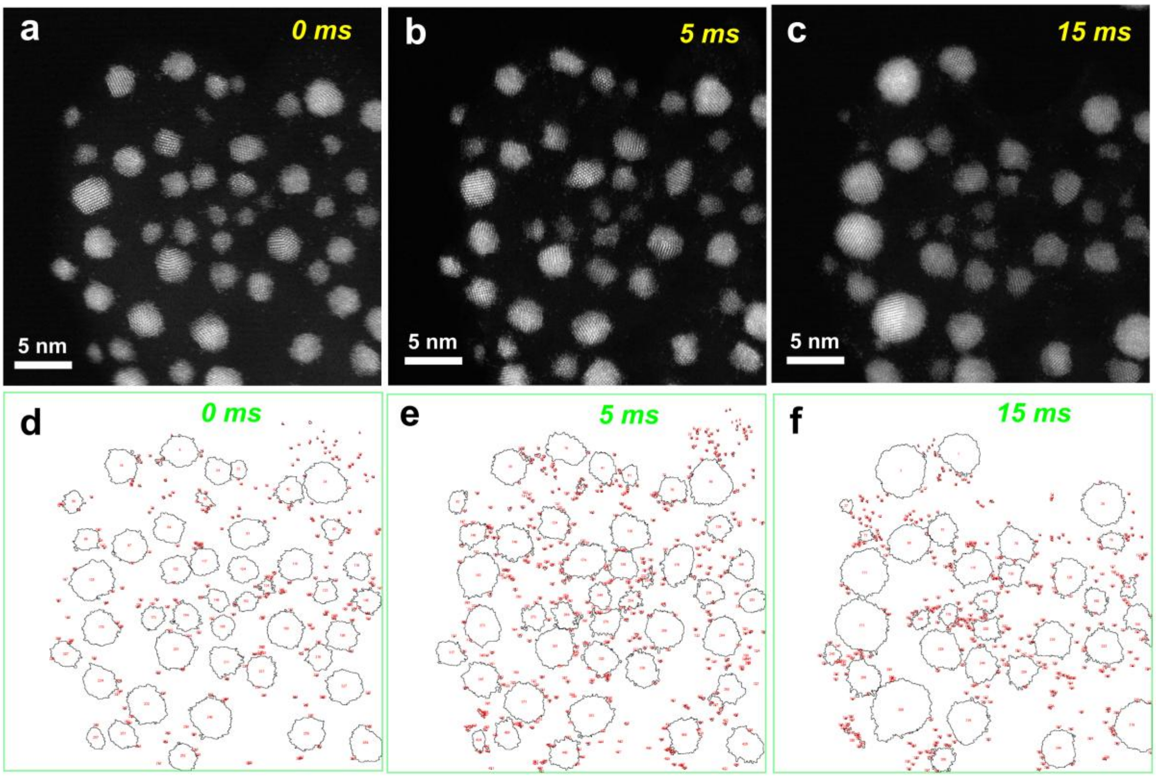

Figure S10. (a-c) The raw IL-ADF-STEM images of gold NPs and single atoms for anodic dissolution times of 0, 5 ms and 15 ms. (d-f) The outlines of these structures obtained from the raw images (a-c).

Table S1. Total number of single gold atoms and NPs obtained from image analysis at different anodic dissolution times.

	0 ms	5 ms	15 ms
**Single atoms**	228	432	352
**NPs**	44	41	39

SI 8. Another set of data showing segmented IL-ADF-STEM images of gold NPs and atom size changes

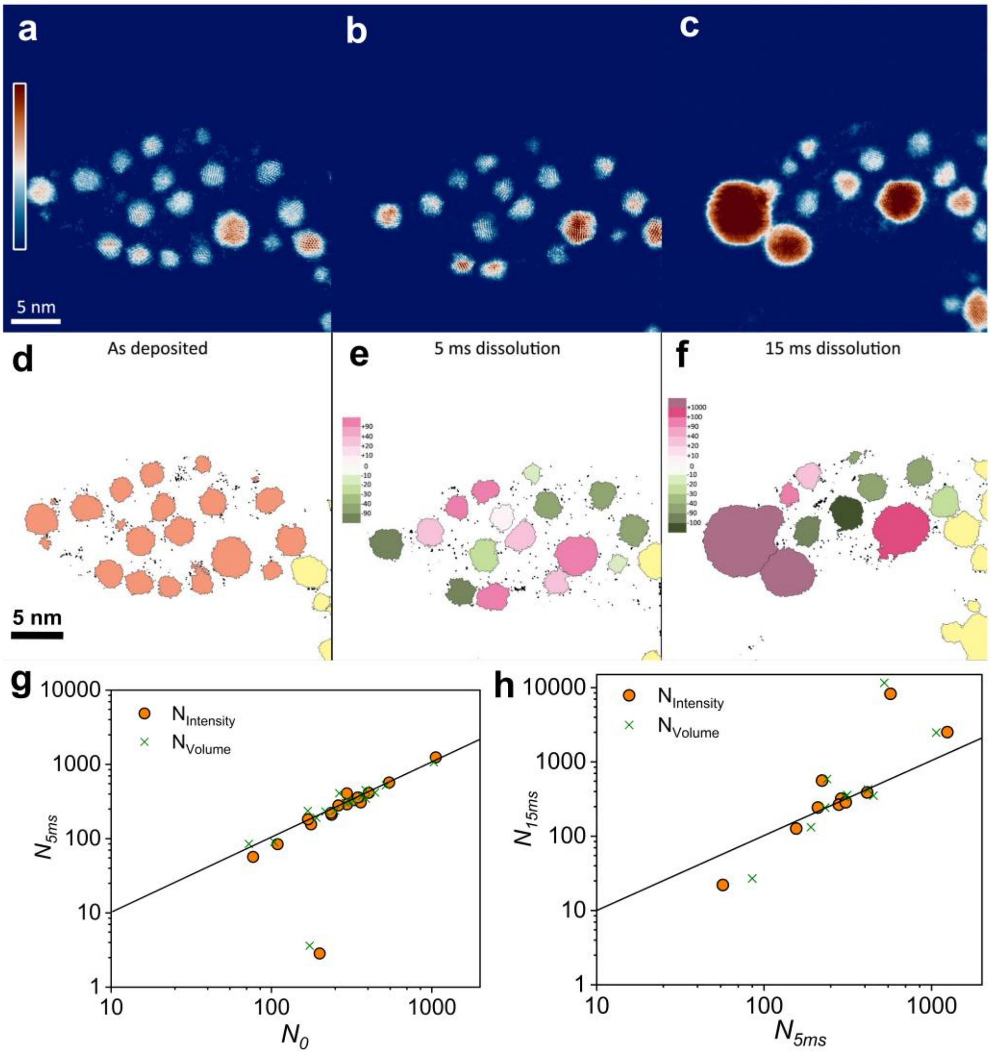

Figure S11. (a-c) The intensity IL-ADF-STEM images of gold NPs with background subtracted. (d-f) The segmented gold particle analysis of IL-ADF-STEM images at one location for the dissolution series. Color coding, as-deposited: orange = counted, yellow = excluded (edge); other images blue = merged, green = shrinking, red = expanding, with contrast proportional to the change in atom count. NPs that disappear completely are shown in grey. (g, h) Scatter plot of atom number changes in each NP over the dissolution series, shown on a logarithmic scale. The black line indicates no change. (g) from as-deposited to 5 ms dissolution and (h) from 5 ms to 15 ms dissolution. Circles = atom count estimated from integrated intensity (method 3) crosses = estimated from (assumed hemispherical) volume (method 2). NPs that disappear or merge with others are shown below the horizontal axis.

SI 9. Estimated numbers of atoms for NPs

Table S2. Estimated numbers of atoms for NPs in Figure 5 (main text) over 0 to 15 ms using the integrated ADF-STEM intensity.

NP number \ Atoms	0 ms<br>N_0	5 ms<br>N_5	15 ms<br>N_{15}
#1	23	9	0
#2	79	37	0
#3	92	80	76
#4	181	85	63
#5	520	624	593
#6	135	150	118
#7	244	238	329
#8	304	307	242
#9	439	432	423

Table S3. Estimated numbers of atoms for NPs in Figure 6 (main text) over 0 to 15 ms using the integrated ADF-STEM intensity.

NP number \ Atoms	0 ms<br>N_0	5 ms<br>N_5	15 ms<br>N_{15}
#10	92	82	23
#11	125	104	22
#12	704	723	1089
#13	416	395	730
#14	275	256	224

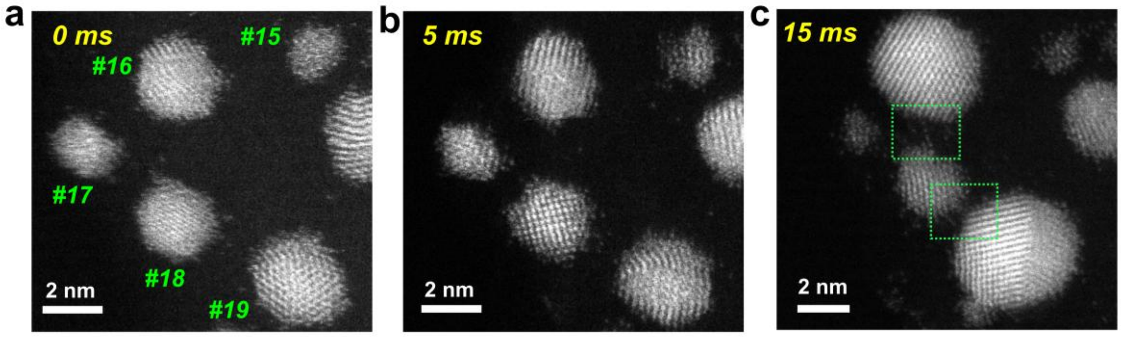

Figure S12. IL-ADF-STEM "time-stamped" images of gold NP behaviors during electrochemical dissolution over 0 to 15 ms. Electrochemical dissolution was carried out by applying + 1.4 V vs Ag/AgCl in 4 mM chloride solution for (a) 0 ms, (b) 5 ms, and (c) 15 ms. Green rectangles indicate atom bridges.

Table S4. Estimated numbers of atoms for NPs in Figure S12 over 0 to 15 ms using the integrated ADF-STEM intensity.

Atoms / NP number	0 ms N_0	5 ms N_5	15 ms N_{15}
#15	132	118	53
#16	492	508	1573
#17	224	224	54
#18	397	412	388
#19	572	560	2247

SI 10. Possible impacts of OCP on NP growth

To investigate the impact of open circuit potential (OCP) on NP growth, the OCP of gold NPs on a BDD macroelectrode was measured before and after 15 ms electrochemical dissolution at + 1.4 V vs Ag/AgCl in 4 mM KCl and 0.1 M $HClO_4$. As shown in Figure S13, the OCP stabilizes to ~0.43 V vs Ag/AgCl before and after dissolution.

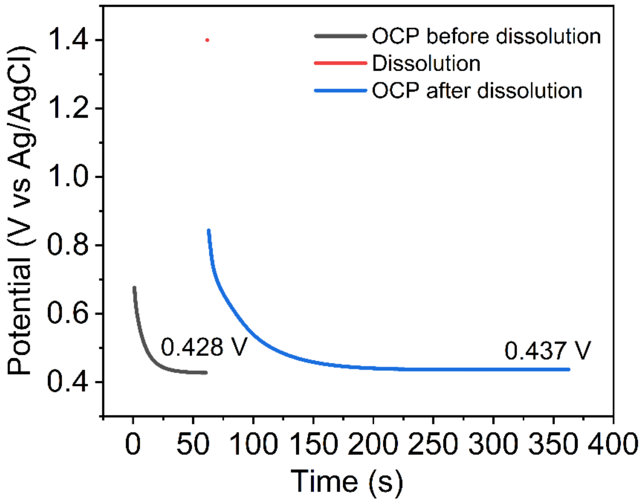

Figure S13. OCP measurements of gold NPs before and after 15 ms electrochemical dissolution on a BDD macroelectrode in 4 mM KCl and 0.1 M $HClO_4$.

At this OCP, it is conceivable that based on the CV response in Figure 2 (main text) that gold electrodeposition could be promoted as a result of the dissolved gold ions in solution. An OCP control experiment was performed where the gold NP-BDD TEM substrate was placed into a solution containing the original solution of 4 mM KCl and 0.1 M $HClO_4$ solution, in the presence of 10 nM $AuCl_4^-$, for 1 min and then removed. 10 nM was used as an estimate of the dissolved gold concentration based on inductively coupled plasma mass spectrometer (ICP−MS) data (Table S5).

Table S5. Summary of the dissolved gold concentration at different electrochemical dissolution time periods, obtained from ICP-MS data (solution volume ~15 mL).

	**0-5 ms**	**5-15 ms**
**Concentration (ppb)**	2.09	1.39

ICP-MS Measurements were conducted in an Agilent 7900 instrument, equipped with SPS4 Auto-sampler with an internal standard (Er 1ppb). The plasma conditions are as followed: plasma gas: 15 L/min, carrier gas: 0.9 L/min, Nebulizer gas: 1.05 L/min, Auxiliary gas: 0.9 L/min, Spray chamber temperature: 15°C, Helium mode gas flow 4.0 ml/min, Forward power 1550W, flow rate: 0.1rps, detector analogue HV 2152V, pulse HV 956V, Discriminator 5 mV. Instrument is auto-tuned before sequence and the P/A factor adjusted using calibration standards during analysis. The instrument was calibrated with gold standards in a matching background in the concentration range 0-1000 ppb. The dissolved gold concentration at two dissolution time period (0-5 ms and 5-15 ms) was measured based on ~15 mL solution and the results are shown in Table S5.

IL-ADF-STEM images were recorded before and after this control experiment, as shown in Figure S14. Visually, in the areas within the green rectangles there is evidence of coalescence / NP rearrangement, but this is limited. However, it means we cannot rule out a possible role for OCP in the growth behavior observed.

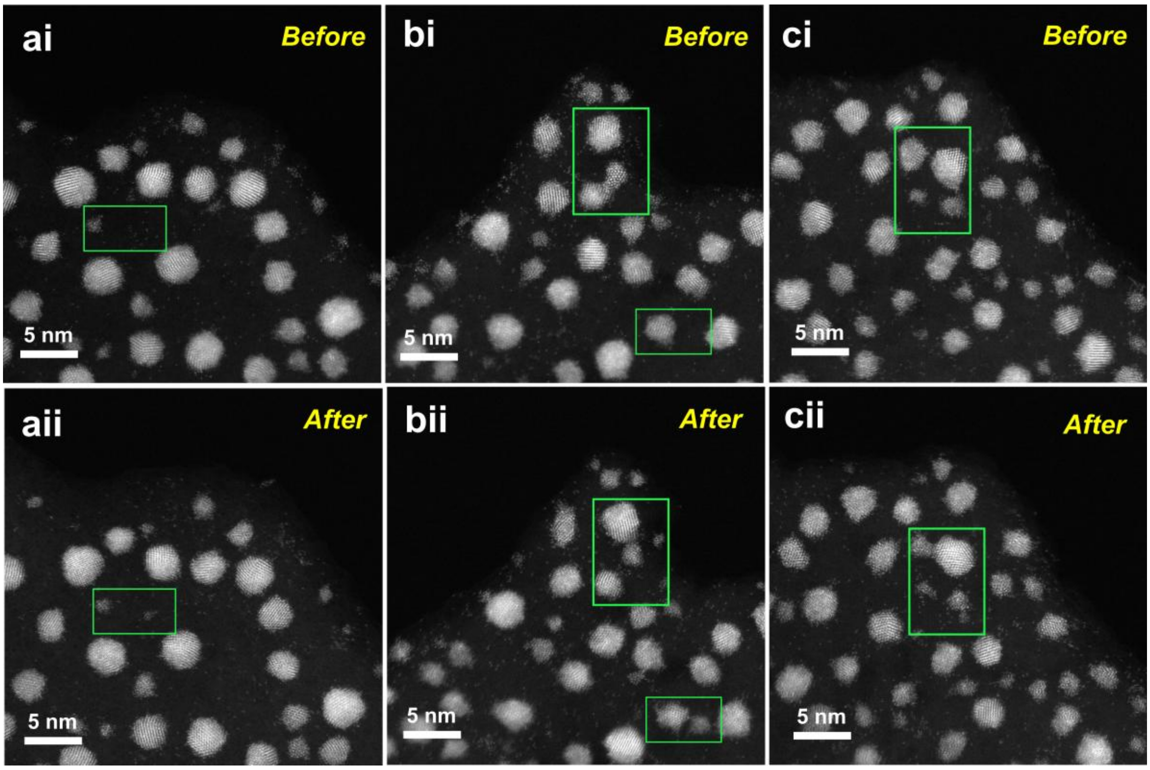

Figure S14. IL-ADF-STEM images of gold NPs on the BDD TEM substrate before and after the OCP control experiment from three different locations (a, b and c). The OCP control experiment was conducted in 10 nM $AuCl_4^-$, 4 mM KCl and 0.1 M $HClO_4$ solution for 1 min, without any potential applied.

SI 11. Further examples of gold NP coalescence

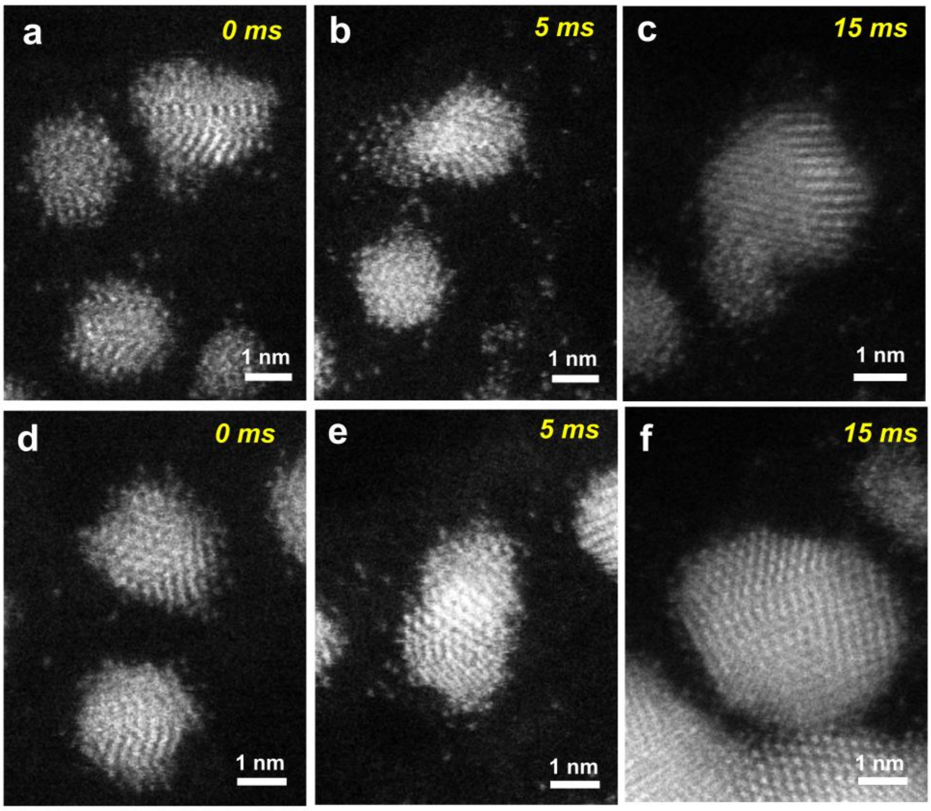

Figure S15. IL-ADF-STEM images showing coalescence of gold NPs during electrochemical dissolution over 0 to 15 ms from two different locations (a-c) and (d-f). Electrochemical dissolution was carried out by applying + 1.4 V vs Ag/AgCl in 4 mM KCl and 0.1 M $HClO_4$.

References

[1] L. A. Hutton, J. G. Iacobini, E. Bitziou, R. B. Channon, M. E. Newton, J. V Macpherson, "Examination of the Factors Affecting the Electrochemical Performance of Oxygen-Terminated Polycrystalline Boron-Doped Diamond Electrodes" *Anal. Chem.* **2013**, *85*, 7230–7240.

[2] H. E. M. Hussein, G. Wood, D. Houghton, M. Walker, Y. Han, P. Zhao, R. Beanland, J. V Macpherson, "Electron Beam Transparent Boron Doped Diamond Electrodes for Combined Electrochemistry─Transmission Electron Microscopy" *ACS Measurement Science Au* **2022**, *2*, 439–448.

[3] S. J. Cobb, F. H. J. Laidlaw, G. West, G. Wood, M. E. Newton, R. Beanland, J. V Macpherson, "Assessment of acid and thermal oxidation treatments for removing sp2 bonded carbon from the surface of boron doped diamond" *Carbon N. Y.* **2020**, *167*, 1–10.

[4] L. Hutton, Mark. E. Newton, P. R. Unwin, J. V Macpherson, "Amperometric Oxygen Sensor Based on a Platinum Nanoparticle-Modified Polycrystalline Boron Doped Diamond Disk Electrode" *Anal. Chem.* **2009**, *81*, 1023–1032.

[5] T. Mori, T. Hegmann, "Determining the composition of gold nanoparticles: a compilation of shapes, sizes, and calculations using geometric considerations" *Journal of Nanoparticle Research* **2016**, *18*, 295.

[6] R. C. G. Killean, E. J. Lisher, "The Debye temperatures of the face centred cubic metals. I. X-ray and neutron diffraction results" *Journal of Physics F: Metal Physics* **1975**, *5*, 1107.

[7] H. E. M. Hussein, G. Wood, D. Houghton, M. Walker, Y. Han, P. Zhao, R. Beanland, J. V Macpherson, "Electron Beam Transparent Boron Doped Diamond Electrodes for Combined Electrochemistry─Transmission Electron Microscopy" *ACS Measurement Science Au* **2022**, *2*, 439–448.

[8] J. Mayer, L. A. Giannuzzi, T. Kamino, J. Michael, "TEM Sample Preparation and FIB-Induced Damage" *MRS Bull.* **2007**, *32*, 400–407.

[9] H. E. M. Hussein, R. J. Maurer, H. Amari, J. J. P. Peters, L. Meng, R. Beanland, M. E. Newton, J. V Macpherson, "Tracking Metal Electrodeposition Dynamics from Nucleation and Growth of a Single Atom to a Crystalline Nanoparticle" *ACS Nano* **2018**, *12*, 7388–7396.
